# Further Evidence of Subphenotype Association with Systemic Lupus Erythematosus Susceptibility Loci: A European Cases Only Study

**DOI:** 10.1371/journal.pone.0045356

**Published:** 2012-09-26

**Authors:** Elisa Alonso-Perez, Marian Suarez-Gestal, Manuel Calaza, Josep Ordi-Ros, Eva Balada, Marc Bijl, Chryssa Papasteriades, Patricia Carreira, Fotini N. Skopouli, Torsten Witte, Emöke Endreffy, Maurizio Marchini, Sergio Migliaresi, Gian Domenico Sebastiani, Maria Jose Santos, Ana Suarez, Francisco J. Blanco, Nadia Barizzone, Rudolf Pullmann, Sarka Ruzickova, Bernard R. Lauwerys, Juan J. Gomez-Reino, Antonio Gonzalez, European Consortium of SLE DNA Collections

**Affiliations:** 1 Laboratorio de Investigacion 10 and Rheumatology Unit, Instituto de Investigacion Sanitaria - Hospital Clinico Universitario de Santiago, Santiago de Compostela, Spain; 2 Internal Medicine, Research Laboratory in Autoimmune Diseases, Hospital Vall d’Hebron, Barcelona, Spain; 3 Department of Internal Medicine and Rheumatology, Martini Hospital, Groningen, The Netherlands; 4 Department of Histocompatibility and Immunology, Evangelismos Hospital, Athens, Greece; 5 Rheumatology Department, Hospital 12 de Octubre, Madrid, Spain; 6 Pathophysiology Department, Athens University Medical School, Athens, Greece; 7 Division of Clinical Immunology, Department of Internal Medicine of the Hannover Medical School, Hannover, Germany; 8 Paediatrics Department, Albert Szent-Györgyi Medical Centre, University of Szeged, Szeged, Hungary; 9 Referral Center for Systemic Autoimmune Diseases, Fondazione IRCCS Ca’ Granda Ospedale Maggiore Policlinico and University of Milan, Milan, Italy; 10 Rheumatology Unit, Second University of Naples, Naples, Italy; 11 UOC Reumatologia, Azienda Ospedaliera San Camillo-Forlanini, Roma, Italy; 12 Rheumatology Department, Hospital Garcia de Orta and Rheumatology Research Unit, Instituto Medicina Molecular, Lisboa, Portugal; 13 Department of Functional Biology, Hospital Universitario Central de Asturias, Universidad de Oviedo, Oviedo, Spain; 14 INIBIC-CH Universitario A Coruña, A Coruña, Spain; 15 Department of Medical Sciences and IRCAD, Eastern Piedmont University, Novara, Italy; 16 Institute of Clinical Biochemistry, Martin Faculty Hospital, Jessenius Medical Faculty, Martin, Slovakia; 17 Institute of Biotechnology, Academy of Sciences of the Czech Republic, Prague, Czech Republic; 18 Cliniques Universitaires Saint-Luc and Université Catholique de Louvain, Brussels, Belgium; 19 Department of Medicine, University of Santiago de Compostela, Santiago de Compostela, Spain; Kunming Institute of Zoology, Chinese Academy of Sciences, China

## Abstract

**Introduction:**

Systemic Lupus Erythematosus (SLE) shows a spectrum of clinical manifestations that complicate its diagnosis, treatment and research. This variability is likely related with environmental exposures and genetic factors among which known SLE susceptibility loci are prime candidates. The first published analyses seem to indicate that this is the case for some of them, but results are still inconclusive and we aimed to further explore this question.

**Methods:**

European SLE patients, 1444, recruited at 17 centres from 10 countries were analyzed. Genotypes for 26 SLE associated SNPs were compared between patients with and without each of 11 clinical features: ten of the American College of Rheumatology (ACR) classification criteria (except ANAs) and age of disease onset. These analyses were adjusted for centre of recruitment, top ancestry informative markers, gender and time of follow-up. Overlap of samples with previous studies was excluded for assessing replication.

**Results:**

There were three new associations: the SNPs in *XKR6* and in *FAM167A-BLK* were associated with lupus nephritis (OR = 0.76 and 1.30, *P_corr_* = 0.007 and 0.03, respectively) and the SNP of *MECP2*, which is in chromosome X, with earlier age of disease onset in men. The previously reported association of STAT4 with early age of disease onset was replicated. Some other results were suggestive of the presence of additional associations. Together, the association signals provided support to some previous findings and to the characterization of lupus nephritis, autoantibodies and age of disease onset as the clinical features more associated with SLE loci.

**Conclusion:**

Some of the SLE loci shape the disease phenotype in addition to increase susceptibility to SLE. This influence is more prominent for some clinical features than for others. However, results are only partially consistent between studies and subphenotype specific GWAS are needed to unravel their genetic component.

## Introduction

Systemic Lupus Erythematosus shows a wide spectrum of clinical manifestations and laboratory findings that complicate its diagnosis, treatment and research [1], [2]. We do not know the causes of this variability, but likely contributing elements are environmental exposures and genetic factors. Understanding the relationships between these factors and the SLE clinical features will help elucidate disease mechanisms and could provide the basis for a classification of patients in more homogeneous subgroups.

Many associations between genetic polymorphisms and the presence of specific SLE clinical features have been reported, but they have not yet reached a high degree of confidence [3], [4], [5]. This situation is similar to what had happened in relation to SLE susceptibility until 2008 when the first SLE GWAS were published. Now, there are about 30 loci that are consistently associated with SLE susceptibility [6], [7], [8]. These loci are prime candidates for having a predisposing effect for specific SLE clinical features. The first analyses seem to indicate that this is the case for some of them [9], [10], [11], [12], [13], [14], [15], but results are still inconclusive. For example, the *rs7574865* SNP in *STAT4* has been associated with a severe SLE phenotype defined by nephritis, age at diagnosis <30 years old, immunologic disorder (and, specifically, double-stranded DNA autoantibodies) and absence of oral ulcers [10]. The association with immunologic disorder (and anti-dsDNA) and an increase in nephritis prevalence were also reported in two other studies, but not the associations with early onset or lack of oral ulcers [9], [13]. In contrast, a fourth study has reported protection from oral ulcers but not association with any of the other subphenotypes [14].

Herein, we have analyzed the association with SLE clinical features of 26 SNPs that tag SLE susceptibility loci in 1444 European patients. Significant associations of renal involvement with *XKR6* and *FAM167A-BLK* SNPs and of earlier age of disease onset and the *MECP2* SNP in men were found. In addition, other weak signals of association were detected that together with concordant previous results obtained in non-overlapping samples suggest a wider involvement of the susceptibility loci in shaping some aspects of the SLE phenotype.

## Materials and Methods

### Ethic Statement

This study was conducted according to the principles expressed in the Declaration of Helsinki. All subjects provided their written informed consent as approved by the respective ethical committees and the overall study was approved by the Comite Etico de Investigacion Clinica de Galicia.

### Clinical and Genotype Data

Patients used in this study have already been described [16], [17]. Briefly, 17 recruiting centres from 10 European countries were asked for about 100 SLE patients according with ACR criteria [18], all of them with uniform ancestry from the country of recruitment. Clinical characteristics of the patients were obtained at the same time. They included the ACR classification criteria, gender, age of disease onset, and time of follow-up. Genotypes of 26 SNPs tagging SLE loci indentified in large studies [19], [20], [21], [22], [23], [24], [25], [26], [27], [28] were available to us from previous studies [16], [29], [30]. They include 2 SNPs tagging the risk and the protective haplotypes of *IRF5* [29], 10 tag SNPs for 10 SLE loci [16], and the 14 tag SNPs in 12 SLE loci [30]. In addition, genotypes of six (rs6730157, rs382259, rs4988235, rs12203592, rs354690 and rs12913832) top ancestry informative markers (AIM) have also been obtained previously [17]. The three first are the most informative AIMs in differentiating Northern from Southern European subpopulations, and rs12203592 and rs354690 are the two AIMs more informative for East-West differentiation, identified in a study analyzing 300 000 SNPs in 4000 European subjects [31]. Results from rs4988235 were not used for analysis because it was largely redundant with rs6730157 in our samples (r^2^ = 0.87). rs12913832 is a SNP associated with large differences in frequency across Europe and unrelated with the previous [32].

### Association Analysis

We compared SNP genotype frequencies between cases positive and negative for each of the ACR classification criteria (except presence of ANA given that they were almost uniformly positive) and age of disease onset. Only patients with information of at least 9 of these 11 features were included in the study (Table 1). The comparisons were done with logistic regression following a genetic additive model with codes 2 for aa genotypes, 1 for aA genotypes and 0 for the AA genotypes. Possible confounding factors were included as covariates in the regression model. These included the 17 recruiting centres as dichotomous variables with value 1 if the sample came from that centre and 0 otherwise. We added also as covariates the patient genotypes at the five top AIMs. In addition, a variable with codes 1 for samples from the Iberian Peninsula and 0 from the rest of samples was added for the analysis of oral ulcers because this is an important differential factor for this clinical feature [30]. Results with P<0.05 at this stage were further analyzed by stratifying by patient gender and by including as covariate time of follow-up. Bonferroni correction for multiple tests was applied considering the number of analyzed clinical features. To compare our results with previous reports, samples from collections that have participated in any of them were excluded. These analyses were done in a customized version of Statistica 7.0 (StatSoft, Tulsa, OK, USA).

**Table 1:** Clinical characteristics of the patients with SLE included in the analyses.

Features	value	% available information
Women (%)	90.5	99.4
Age at disease onset (mean, SD)	30.7 (13.0)	95.8
Time of follow-up (mean, SD)	12.0 (8.4)	83.5
Malar rash (%)	56.0	93.8
Discoid rash (%)	17.7	99.5
Photosensitivity (%)	52.2	99.9
Oral ulcers (%)	27.4	99.9
Arthritis (%)	79.0	100.0
Serositis (%)	35.3	99.9
Renal disorder (%)	41.6	100.0
Neurologic disorder (%)	14.6	94.0
Hematologic disorder (%)	72.9	97.1
Immunologic disorder (%)	77.9	99.5

## Results

We collected DNA samples and clinical information from 1742 patients with SLE from 17 recruiting centres in 10 European countries. They were ascertained to have a uniform self-reported origin from the country of recruitment and genotyped for top AIMs informative about European population substructure. We required for inclusion in this study that each patient should have complete genotypes for the top AIMs and almost complete clinical information defined as 9 or more of the following data: ACR classification criteria, gender and age of disease onset. These conditions were fulfilled for 1444 patients (Table 1). We had already genotyped these samples for 26 SNPs tagging independent SLE association signals [16], [29], [30], which have been identified in other large studies (Table 2). All of them have passed our quality control filters and most of them were significantly associated in our samples and showed the same direction of change and a very similar effect size than the reported in the study where they were discovered (Table 2; and Table S1 for individual genotypes). There were only 5 of these SNPs that were non-associated in our samples.

**Table 2:** SNPs tagging SLE susceptibility loci with indication of their association in the study where they were discovered and in our current samples [16], [29], [30].

		SNP discovery study			Current study	
SNP	Locus	OR (95% CI)	*P*	Ref.	OR (95% CI)	*P*
*rs2187668*	*HLA-DQA1*	1.9 (1.7–2.2)	3.0×10^−21^	22	2.2 (1.9–2.5)	1.1×10^−25^
*rs10488631*	*IRF5*	1.7 (1.5–1.9)	1.7×10^−11^	22	2.0 (1.7–2.3)	8.4×10^−21^
*rs3131379*	*MSH5*	2.4 (2.1–2.6)	1.7×10^−52^	21	2.3 (1.9–2.7)	6.4×10^−20^
*rs1143679*	*ITGAM*	1.8 (1.6–2.0)	1.7×10^−17^	25	1.7 (1.5–1.9)	1.1×10^−16^
*rs7574865*	*STAT4*	1.5 (1.2–1.8)	2.8×10^−9^	21	1.6 (1.4–1.9)	2.4×10^−12^
*rs2230926*	*TNFAIP3*	2.0 (1.4–3.0)	3.0×10^−4^	24	2.0 (1.6–2.5)	2.5×10^−10^
*rs729302*	*IRF5*	0.7 (0.5–0.8)	6.7×10^−3^	19	0.7 (0.7–0.8)	1.7×10^−7^
*rs13277113*	*FAM167A-BLK*	1.4 (1.3–1.5)	1.1×10^−10^	22	1.3 (1.2–1.5)	5.1×10^−7^
*rs2304256*	*TYK2*	0.6 (0.5–0.8)	5.6×10^−5^	19	0.8 (0.7–0.9)	2.5×10^−5^
*rs5754217*	*UBE2L3*	1.2 (1.1–1.3)	7.5×10^−8^	21	1.3 (1.1–1.4)	7.3×10^−5^
*rs10798269*	*1q25.1*	0.8 (0.8–0.9)	1.1×10^−7^	21	0.8 (0.7–0.9)	1.3×10^−4^
*rs2205960*	*TNFSF4*	1.3 (1.1–1.5)	7.0×10^−3^	20	1.3 (1.1–1.4)	1.3×10^−4^
*rs844644*	*TNFSF4*	0.7 (0.6–0.8)	6.8×10^−5^	20	0.8 (0.7–0.9)	7.2×10^−4^
*rs17435* a	*MECP2*	1.3 (1.1–1.5)	8.0×10^−4^	26	1.3 (1.1–1.4)	8.5×10^−4^
*rs2476601*	*PTPN22*	1.5 (1.3–1.8)	5.2×10^−6^	21	1.3 (1.1–1.6)	9.2×10^−4^
*rs4963128*	*KIAA1542*	0.8 (0.7–0.9)	3.0×10^−10^	21	0.8 (0.8–0.9)	1.1×10^−3^
*rs1801274*	*FCGRIIA*	1.4 (1.2–1.5)	6.8×10^−7^	21	1.2 (1.1–1.3)	1.3×10^−3^
*rs6920220*	*TNFAIP3*	1.2 (1.1–1.3)	4.0×10^−7^	27	1.2 (1.1–1.4)	2.3×10^−3^
*rs6445975*	*PXK*	1.3 (1.2–1.4)	7.1×10^−10^	21	1.2 (1.1–1.3)	3.8×10^−3^
*rs573775*	*ATG5*	1.2 (1.1–1.3)	1.4×10^−7^	21	1.2 (1.1–1.3)	4.4×10^−3^
*rs17266594*	*BANK1*	0.7 (0.6–0.8)	4.7×10^−11^	23	0.8 (0.7–0.9)	6.2×10^−3^
*rs4240671*	*XKR6*	0.75 (0.7–0.8)	6.6×10^−9^	21	0.9 (0.8–1.0)	0.11
*rs10156091*	*ICA1*	1.3 (1.2–1.5)	1.9×10^−7^	21	1.1 (0.9–1.3)	0.3
*rs2667978*	*LYN*	0.8 (0.7–0.8)	5.1×10^−8^	21	0.9 (0.8–1.1)	0.3
*rs509749*	*LY9*	377∶403 b	2.1×10^−3^	28	1.0 (0.9–1.1)	0.5
*rs6922466*	*PERP*	0.8 (0.7–0.9)	1.0×10^−4^	24	1.0 (0.9–1.1)	0.8

OR: Odds ratio. CI: confidence interval.

a Only women. The result for men in our samples was O.R. = 2.11 (1.3–3.4), *P* = 0.001.

b Oberved:Expected in transmission disequilibrium test.

In the current case-only analysis, we compared genotype frequencies of each of the SLE SNPs between SLE patients showing the clinical features with those not showing them. All the analyses were adjusted for recruiting centre and for the five top non-redundant AIMs. This is necessary because some of the features and some of the SLE risk alleles are known to vary between European subpopulations [17], [30], [33], [34], [35].

Renal disorder was the clinical feature showing more association signals (Table 3). It was associated with the *XKR6*, *FAM167A-BLK*, *TNFSF4* and *ICA1* SNPs. Only the effect of *FAM167A-BLK* was of increased risk with the SLE risk allele. In all the others the risk allele was protective for nephritis. All these associations persisted after adjusting for gender and time of follow-up (Table S2) and the two first, with *XKR6* and *FAM167A-BLK* SNPs, were significant after correction by the number of tests (Table 3).

Age of disease onset, either as a dichotomous or as a continuous variable, was also nominally associated with three SLE loci (Table 3). There was association with age at onset with the *ATG5*, *STAT4* and *MECP2* SNPs. The association with *MECP2* was the unique association over the threshold for multiple tests. However, special caution with this result is required because it was observed only in men. They were analyzed separately from women because this locus is in the X chromosome. The risk allele of *ATG5* was associated with older age of onset and the risk alleles of the other two loci with younger onset. These association signals did not improve by considering age of onset in its full quantitative range with linear regression (Table 3 and *rs573775* in *ATG5*, P = 0.3). The association of *STAT4* with earlier SLE onset had already been reported and with a very similar odds ratio (OR) in a study that did not include overlapping samples with the current one [10].

None of the remaining association signals obtained with other clinical features was over the threshold accounting for multiple tests (Table 3). Among the weak association signals there were three with immunological disorder: *TNFSF4*, *LY9* and *XKR6*. Neurologic disorder was nominally associated with SNPs in *IRF5* and *LY9*, serositis with SNPs in *LYN* and *TYK2*, discoid rash with SNPs in *IRF5* and *TYK2*, photosensitivity with SNPs in *FCGR2A* and *TNFSF4*, hematologic disorder with the SNP in *MSH5*, and oral ulcers with the *PTPN22* nsSNP. None of these nominal associations has previously been reported. In addition, we did not replicate any of the other previously reported associations beyond the already commented of *STAT4* with early disease onset (Table 4). However, some of our results were concordant in direction and magnitude as between the *TNFSF4* SNP and lupus nephritis [14], and *STAT4* SNP and protection from oral ulcers or risk to immunologic disorder [10], [14].

**Table 3:** SLE clinical characteristics showing association with SLE susceptibility SNPs.

Feature	SNP	Locus	*P*	OR (95% CI)	*P_corr_*
Renal disorder	*rs4240671*	*XKR6*	0.0006	0.76 (0.7–0.9)	0.007
	*rs13277113*	*FAM167A-BLK*	0.003	1.30 (1.1–1.5)	0.03
	*rs10156091*	*ICA1*	0.03	0.76 (0.6–1.0)	0.3
	*rs844644*	*TNFSF4*	0.04	0.85 (0.7–1.0)	0.4
Diseaseonset <30	*rs573775*	*ATG5*	0.005	0.78 (0.7–0.9)	0.06
	*rs7574865*	*STAT4*	0.03	1.20 (1.0–1.4)	0.3
	*rs17435*	*MECP2* a	0.003	1.92 (1.2–3.0)	0.03
Disease onsetfull range	*rs7574865*	*STAT4*	0.02	−1.21 (−2.2 −0.2)^b^	0.2
	*rs17435*	*MECP2* a	0.02	−3.57 (−6.6 −0.5)^b^	0.3
Immunologic disorder	*rs2205960*	*TNFSF4*	0.01	1.39 (1.1–1.8)	0.1
	*rs509749*	*LY9*	0.01	1.31 (1.1–1.6)	0.2
	*rs4240671*	*XKR6*	0.04	0.81 (0.7–1.0)	0.5
Neurologic disorder	*rs10488631*	*IRF5*	0.01	0.64 (0.5–0.9)	0.1
	*rs509749*	*LY9*	0.04	1.26 (1.0–1.6)	0.4
Serositis	*rs2667978*	*LYN*	0.02	0.79 (0.7–1.0)	0.2
	*rs2304256*	*TYK2*	0.03	1.23 (1.0–1.5)	0.3
Discoid rash	*rs729302*	*IRF5*	0.02	0.75 (0.6–1.0)	0.2
	*rs2304256*	*TYK2*	0.03	1.29 (1.0–1.6)	0.3
Photosensitivity	*rs1801274*	*FCGRIIA*	0.03	1.19 (1.0–1.4)	0.3
	*rs2205960*	*TNFSF4*	0.03	1.22 (1.0–1.5)	0.3
Hematologic disorder	*rs3131379*	*MSH5*	0.03	1.37 (1.0–1.8)	0.3
Oral ulcers	*rs2476601*	*PTPN22*	0.04	0.72 (0.5–1.0)	0.5

a Men only.

b Coefficient of the linear regression.

## Discussion

Our study has found that some of the SLE susceptibility loci contribute to shape the SLE phenotype. In addition, it has shown more associations with some clinical features than with others, with a pattern that is reproducible and that indicates the most productive SLE subphenotypes for future projects. Some of the specific associations start to be consistently observed in different studies, although our results are not strong enough to firmly establish them. Three of the associations we have found were over the threshold accounting for multiple tests and one replicated a previous finding. Some of the remaining associations were also of interest because they were concordant in direction and magnitude with previous reports.

The clearest associations were found between renal disorder and SNPs in the *XKR6* and *FAM167A-BLK* loci. Both associations remained significant after correction for multiple tests. *XKR6* was found associated with SLE in the SLEGEN GWAS with 5 SNPs showing P<5×10^−8^, although it was not highlighted because they were not uniformly associated in the 4 sets of samples included in that study [21]. There has been independent replication of one of the SNPs in a subsequent study [36]. The SNP reported here was not associated with SLE in our samples (Table 2), and none of the *XKR6* SNPs were associated in the largest attempt to replicate SLE GWAs results [27]. Therefore the status of this locus in SLE susceptibility is still uncertain. We are not aware of any previous attempt to explore association between SNPs in this gene and SLE clinical features. The *XKR6* gene codes for one of the transmembrane proteins of the Kell blood group of antigens expressed in red blood cells, although it is also expressed in other tissues. Its possible role in SLE is completely unclear because almost nothing is known about this protein.

Association between lupus nephritis and the SLE risk allele of *rs13277113* in the *FAM167A-BLK* locus remained significant after correcting for the number of tests, and was consistently observed in women and men. The same SNP has already been analyzed for association with SLE classification criteria in two previous overlapping studies [14], [22], but no association with renal involvement was found. A third overlapping study analyzed a different SNP with the same result [13]. As these previous studies are very large, the association we have found should be considered only as tentative.

**Table 4:** Comparison of previous significant SLE subphenotype associations in case-only analysis with our results regarding the same SNPs.

			Previous studies			Current study	
Feature	SNP	Locus	OR (95% CI)	*P*	Ref.	OR (95% CI)	*P*
Renal disorder	*rs2205960*	*TNFSF4*	1.18 (1.1–1.3)	0.003	14	1.16 (0.9–1.5)^a^	0.2^a^
	*rs1143679*	*ITGAM*	1.39 (1.2–1.7)	0.0003	11	1.13 (0.9–1.4)	0.2
	*rs7574865*	*STAT4*	1.23(1.0–1.5)	0.02	10	0.91 (0.8–1.1)	0.3
	*rs2187668*	*HLA-DQA1*	1.37 (1.1–1.6)	0.0006	13	1.03 (0.9–1.2)	0.8
Disease onset <30	*rs7574865*	*STAT4*	1.22 (1.0–1.4)	0.02	10	1.20 (1.0–1.4)	0.03
Immunologic disorder	*rs7574865*	*STAT4*	1.24 (1.0–1.5)	0.02	10	1.16 (0.9–1.5)	0.2
	*rs1143679*	*ITGAM*	1.30 (1.0–1.7)	0.04	11	1.10 (0.8–1.4)	0.5
	*rs4963128*	*KIAA1542*	0.79 (0.7–0.9)	0.002	13	0.98 (0.8–1.2)	0.9
Oral ulcers	*rs7574865*	*STAT4*	0.80 (0.7–0.9)	0.009	10	0.84 (0.7–1.0)	0.07
Malar rash	*rs1801274*	*FCGRIIA*	1.14 (1.0–1.3)	0.01	14	1.10 (0.9–1.3)^a^	0.4 a
Hematologic disorder	*rs13277113*	*FAM167A-BLK*	1.23 (1.0–1.5)	0.02	22	1.07 (0.9–1.3)	0.5
Discoid rash	*rs1143679*	*ITGAM*	1.27 (1.0–1.6)	0.02	11	1.01 (0.8–1.3)	0.9

a OR (95% CI) and *P* values calculated excluding patients from The Netherlands, Germany, Belgium, Hungary, Asturias (Spain), Rome (Italy) and Naples (Italy) which overlapped with the used in [14] under the BIOLUPUS collection.

The third significant association in our study was found between earlier age at disease onset and the risk allele of the *MECP2* SNP. This association should be taken with special caution because it was restricted to men and there were only 137 men with SLE among our patients.

Other signals of association were found, but none was significant after correction for multiple tests. Overall the associations seem to concentrate in some clinical features: renal disorder, age at onset and immunological disorder are the clearest. In contrast, there are clinical phenotypes that did not appear in any analysis, like malar rash or arthritis. This is in agreement with previous reports where also renal disorder, different types of autoantibodies and age of disease onset are prominent [9], [10], [11], [12], [13], [14].

Replication of the association of individual locus with specific SLE phenotypes was only obtained for the previous association between *STAT4* and early age of disease onset [10]. Our study was completely independent from the original study and, therefore, it constitutes formal replication. In addition, our results showed some trends that followed the same direction observed in previous reports. The clearest examples are the associations of a *TNFSF4* SNP with lupus nephritis [14], and the protection conferred by *STAT4* for oral ulcers [10], [14]. For these two comparisons, our results showed the reported direction of change but they were not significant, specially when the samples overlapping between our study and the Sanchez et al. study [14] were excluded. These coincidences are encouraging and they give further motivation for additional studies.

Unfortunately, lack of replication of association results regarding clinical phenotypes is still very common. For example, in two large studies there were 8 claims of association (5 plus 3, respectively), but none was coincident in spite of overlap between the samples [13], [14]. Probably, these results, as well as ours, are reflection of two types of obstacles. The first is related with the studied loci because a weak phenotype specificity of these loci is implied in the fact that they have been identified in studies involving a wide spectrum of patients with SLE. This circumstance makes it likely that many SNPs with association to particular SLE features will be missing from the list of SLE susceptibility loci. This is exemplified by the *FCGR3A* V158F polymorphism that is associated with lupus nephritis, but not with SLE susceptibility overall [37]. Therefore, it is likely that phenotype-specific GWAs will be more informative. As a first step, a reanalysis of the available GWAs stratified by clinical features could provide useful leads. The second type of obstacles is due to the analysis of subphenotypes, which are highly variably between patients, centres and ethnic groups [10], [17], [22], [34], [35], [38], [39], [40], [41]. To account for these factors, we have restricted our analyses to subjects of European ancestry and we have adjusted our results for centre of recruitment and for AIMs informative of population substructure. These are precautions that have not been used uniformly in the studies of this type. However, it is not possible through analysis to recover the lost statistical power due to variability between the collections and division of the available data in strata. These properties of the analysis of subphenotypes are typical of any subgroup analysis and make replication of results more difficult [42].

In spite of the difficulties and that these are still early times for these studies; there are results that start to be consistent. They include the concordances we have already signalled relating *STAT4* with earlier disease onset or oral ulcers [10], [14]. Also, encouraging are the results relative to the association of *ITGAM* with nephritis, which has already been reported in four studies and that showed the same direction in our samples [11], [12], [14], [15]. A clinical characteristic not included in the current study, the production of anti-dsDNA, has also shown reproducible association with some SLE loci [9], [10], [13].

### Conclusion

In summary, we have found some new genetic associations with SLE clinical features among the SLE susceptibility loci. They confirm the hypothesis that some of these loci shape the SLE phenotype in addition to increase susceptibility to the disease. Our results also support a gradation in the clinical features showing association with these loci: with lupus nephritis, immunologic disorder and age of onset showing the most numerous and clear associations. There were three new associations, *XKR6* and *FAM167A-BLK* with lupus nephritis and *MECP2* with early onset of SLE in men, which need to be taken with caution because either they have never been explored before, or they were not found in previous studies. Replication of the association between early age of disease onset and *STAT4* was obtained. Results for other specific associations showed consistency with previous results without amounting to replication. Overall, results in this field show the need to start GWAS specific for SLE subphenotypes either by reanalysis of existing data or through new studies. A prime candidate for these studies is renal disorder because of its clinical relevance and its prominent association with known SLE loci.

## Supporting Information

Table S1 **Individual genotypes for the 26 SNPs tagging SLE loci in the patients included in the study.** Sex, 0 = male; 1 = female; genotypes, 0 = AA, 1 = Aa and 2 = aa.
(XLS)

Table S2 **SLE clinical characteristics showing association with SLE susceptibility SNPs including data adjusted by gender and time of follow-up.** ^a^This result is only for men and therefore the adjustment has been made only for time of follow-up.
(XLS)
